# Neural Induction, Neural Fate Stabilization, and Neural Stem Cells

**DOI:** 10.1100/tsw.2002.217

**Published:** 2002-04-28

**Authors:** Sally A. Moody, Hyun-Soo Je

**Affiliations:** Department of Anatomy and Cell Biology, Genetics Program, Institute for Biomedical Sciences, The George Washington University Medical Center, Washington, DC, USA

**Keywords:** chordin, noggin, follistatin, cerberus, Xnr3, sox2, sox3, zic, Iroquois genes, fox genes, geminin, neurogenin, NeuroD, n-tubulin

## Abstract

The promise of stem cell therapy is expected to greatly benefit the treatment of neurodegenerative diseases. An underlying biological reason for the progressive functional losses associated with these diseases is the extremely low natural rate of self-repair in the nervous system. Although the mature CNS harbors a limited number of self-renewing stem cells, these make a significant contribution to only a few areas of brain. Therefore, it is particularly important to understand how to manipulate embryonic stem cells and adult neural stem cells so their descendants can repopulate and functionally repair damaged brain regions. A large knowledge base has been gathered about the normal processes of neural development. The time has come for this information to be applied to the problems of obtaining sufficient, neurally committed stem cells for clinical use. In this article we review the process of neural induction, by which the embryonic ectodermal cells are directed to form the neural plate, and the process of neural�fate stabilization, by which neural plate cells expand in number and consolidate their neural fate. We will present the current knowledge of the transcription factors and signaling molecules that are known to be involved in these processes. We will discuss how these factors may be relevant to manipulating embryonic stem cells to express a neural fate and to produce large numbers of neurally committed, yet undifferentiated, stem cells for transplantation therapies.

## INTRODUCTION

Interest in stem cell biology has exploded because of its potential application to repair of damaged tissues. Bone marrow stem cells injected into the cardiac wall or mobilized from the host by cytokine treatment can populate and repair damaged heart tissue[92,93]. Hematopoietic stem cells can repopulate the bone marrow and reconstitute blood lineages[63]. Embryonic and cerebral cortex stem cells can be differentiated in culture to produce dopamine neuron precursors that alleviate the symptoms of Parkinson's disease in a mouse model[66,106]. Umbilical cord stem cells can populate damaged brain and improve function after stroke[24]. Furthermore, stem cells from one tissue can be forced into novel differentiated cell types. Muscle stem cells can become muscle cells, but also glial, vascular, bone, blood, and cartilage cells[65]. Blood cells can make brain cells and vice versa[80,129]. This apparent widespread plasticity is both exciting and concerning as we set our sights on human clinical applications. How exciting that we may be able to design protocols to create just about any type of differentiated cell from any tissue. However, if the transplanted cells can be so easily enticed to make different phenotypes, can we be assured they will make the right choices after transplantation? How can we control whether they become muscle when muscle is desired, neurons when neurons are desired, and not some unwanted or detrimental phenotype?

The difficulty of obtaining enough cells with the desired characteristics to implement practical therapies poses a potential problem. In a mouse model for Duscheyne's muscular dystrophy, for example, the protocol for isolating muscle stem cells is tedious and harvests low cell numbers[65]. Since $5 \times 10^5$ cells are required for one experimental injection into one mouse muscle, how many will be needed to help a human patient walk again? At least two fundamental questions must be answered before stem cell transplantation is reliable and efficient. How do we control the differentiation of stem cells to the desired phenotype, and how do we produce enough committed stem cells of the desired phenotype to make clinical applications feasible? In this review we will address these questions with regards to neural stem cells.

The promise of stem cell therapy is expected to have widespread CNS benefits. Neurodegenerative diseases lay waste to the aging human population, resulting in mentally and physically debilitating conditions such as Alzheimer's, Parkinson's, and Huntington's diseases. An underlying biological reason for the progressive losses associated with these diseases is the extremely low natural rate of self-repair in the nervous system. During development, neuroectodermal stem cells complete a terminal mitosis when they begin to differentiate into neurons. When these cells begin to express neuron-specific cytoskeletal genes, sprout axons, and make synapses with target cells, they virtually never divide again (although there are a few exceptions[73]). Scientists have discovered in the past decade that the mature CNS harbors a limited number of self-renewing stem cells[4,32,72], but these make a significant contribution to only a few cerebral areas (e.g., olfactory bulb and hippocampus[19,95]). Regions most affected by neurodegenerative diseases, such as cerebral cortex and basal ganglia, show minimal self-repairing capacity even though stem cells appear present. Despite increased stem cell proliferation after trauma to these regions[54], function recovery is usually slow and incomplete. Therefore, it is particularly important to understand how to manipulate neural stem cells so their descendants can repopulate and functionally repair the affected brain regions. Important information about how to do this is available from studies of the normal processes of neural development. Herein we review the two earliest steps in embryonic neural specification, neural induction, and neural fate stabilization, and discuss how this information might be applied to producing enough neural stem cells of the appropriate fates for transplantation therapies.

### Neural Specification

Specification refers to a developmental program that transduces an initial inductive signal into a differentiated phenotype. In the development of the vertebrate nervous system, this process can be

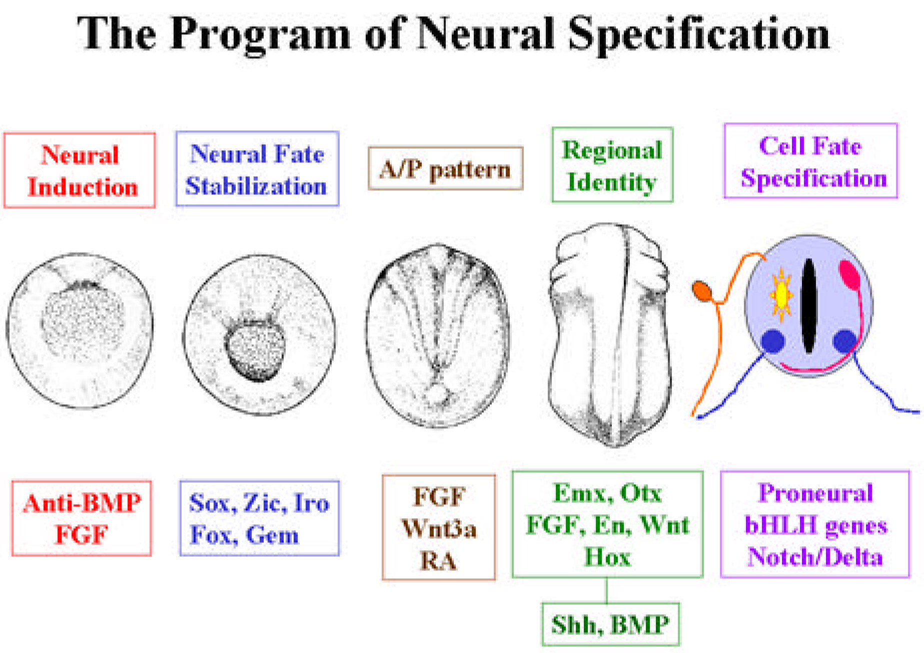

**FIGURE 1.** The developmental program that converts ectoderm to a patterned neural tube, called neural specification, can be divided into several steps. These are named across the top of the panel. Some of the proteins involved in these early steps are boxed along the bottom of the panel. In the middle are representative stages of *Xenopus* development at which these steps are evident, i.e., stage 10 (early gastrula) for neural induction, stage 12 (late gastrula) for neural fate stabilization, stage 13/14 (neural plate) for initial anterior/posterior (A/P) patterning, and stage 23 (closed neural tube) for brain region identity. For the step of cell fate specification, a cross-section of the spinal cord is diagrammed, with different cell types beginning to differentiate (motoneurons, blue; primary sensory neurons, orange; interneurons, red; glia; yellow). Drawings of *Xenopus* embryos from [139].

divided into several distinct steps (Fig. 1). The first, called neural induction, occurs as the embryo goes through gastrulation, the cell movements that organize the vertebrate into three primary germ layers. This event was demonstrated first in amphibian embryos by Spemann and Mangold, who tested whether the presumptive dorsal mesoderm of a gastrulating embryo, called the dorsal lip of the blastopore, could self-differentiate[42,47,111,134]. A transplanted dorsal lip and surrounding host tissues formed neural tube, notochord, and paraxial muscle organized into a secondary embryo with clearly defined axes[111]. These changes were thought to be caused by a signal from the transplant since surrounding host cells also changed fate; thereafter the dorsal lip of the blastopore became known as Spemann's "organizer". The molecular mechanisms of this signal are reviewed in the following section.

Once the domain of neural ectoderm is distinguished from the domain of epidermis, there are several steps that occur to pattern the neural plate and cause the appropriate differentiation of specific cell types at the correct locations of the nervous system (Fig. 1). First, the neural fate of the newly induced ectoderm is stabilized by the expression of a number of genes, to be discussed in detail below, that influence competence and the ability to differentiate into neural derivatives. During this period the neural stem cell population expands, as evidenced by the growth of the neural plate. Next, the neural plate is patterned in both the anterior/posterior and dorsal/ventral axes[36,42]. Region-appropriate phenotypes are controlled by a number of transcription factors and signaling molecules that set up the axes of the brain. Initially the neural plate is entirely "anterior" in character; it expresses genes that later are characteristic of only the anterior part (forebrain, midbrain) of the plate. During gastrulation, signals from the posterior mesoderm, including members of the FGF and

Wnt families and retinoic acid (RA), promote a posterior fate in the adjacent neural plate, thus setting up an initial anterior/posterior difference. One consequence of this anterior-posterior polarity is the patterning of the Hox genes, which will then regulate the segmental identity of the posterior (hindbrain, spinal cord) neural tube. In addition, signaling centers are set up that determine the identity of the rostral brain regions, and the dorsal/ventral polarity of the neural tube is established by opposing sonic hedgehog/BMP gradients[3]. Thus, as neural cells begin to differentiate, they have positional identities that direct which differentiation programs are followed.

Concurrent with the process of axis formation and positional identity acquisition, neural precursor cells begin to express genes that initiate region-specific differentiation programs. In *Drosophila*, neural precursors are specified as two longitudinal stripes of cells by their expression of proneural genes (*achaete, scute,* and *atonal*) that have a basic-helix-loop-helix (bHLH) DNA binding motif[20,114]. Subsequently, elevated expression of *achaete* and lateral inhibition via Notch-Delta signaling isolate more restricted neural precursor cells, called neuroblasts, that then give rise to all the neurons and glia in their segment of the ventral nerve cord. In the fly, proneural genes are the key regulators of the transition between neurally specified stem cells to fate-restricted progenitor cells. Many vertebrate homologues of proneural genes have been isolated and characterized[18,45]. Unlike their fly counterparts, these genes do not visibly act in the earliest steps of neural stem cell specification. In *Xenopus*, for example, the earliest proneural genes (*XASH-3, Xngnr1, XATH3*) are not expressed throughout the neural ectoderm but only in restricted patterns and regulate later expressed differentiation genes. Recent evidence also indicates that the vertebrate proneural genes function in unique combinations to regulate the expression of different cell types[88,122] later in development. Thus, while these genes are certainly critical for cell fate specification, they are not likely involved in regulating the earliest steps of neural stem cells. Therefore, this article focuses on the earliest steps in neural specification, neural induction and neural-fate stabilization.

## NEURAL INDUCTION

Neural induction is a key step in the neural specification program, whose understanding should prove useful in attempts to convert ES cells to neural stem cells. As described above, signals from the “organizer” instruct adjacent embryonic ectoderm to induce the neural tissues of the animal. When neural induction was discovered in the 1920s, the idea that chemical compounds could carry instructions between embryonic regions was novel. Determining the identity of the signaling molecules was impossible given the small tissue source, the low concentration of the molecules in the embryo, and the then-elementary state of biochemical analyses. Nonetheless, many experiments[42] indicated that the neural inductive signal was a diffusible, secreted molecule. The neural-inducing molecules were not identified until recombinant DNA techniques enabled functional screens for rare molecules located in the dorsal lip of the blastopore. These molecules were identified by testing their ability to induce a secondary axis when misexpressed on the ventral side of *Xenopus* embryos. Several secreted molecules were identified that act to induce neural ectoderm by inhibiting the bone morphogenetic protein 4 (BMP4) signaling pathway on the dorsal side of the embryo (Fig. 2; reviewed in detail [29,47,134]). BMP4 induces embryonic ectoderm to become epidermis, and ectoderm not subjected to BMP4 signaling acquires a neural fate. This suggests that the neural fate is the “default” state of embryonic ectoderm[132]. We will discuss five of the neural-inducing molecules: Chordin, Noggin, Follistatin, Cerberus, and Nodal-related-3 (Table 1).

Chordin is expressed prior to gastrulation in the dorsal marginal zone that will later form the dorsal lip. As gastrulation begins, transcripts are located in the dorsal lip and later expressed in the head mesoderm and notochord. In addition to inducing a secondary axis, Chordin dorsalizes mesoderm and induces neural markers in ectodermal explants. Thus, it is located in the correct place and time and causes the appropriate phenotypes to be the Spemann and Mangold “organizer” signal.

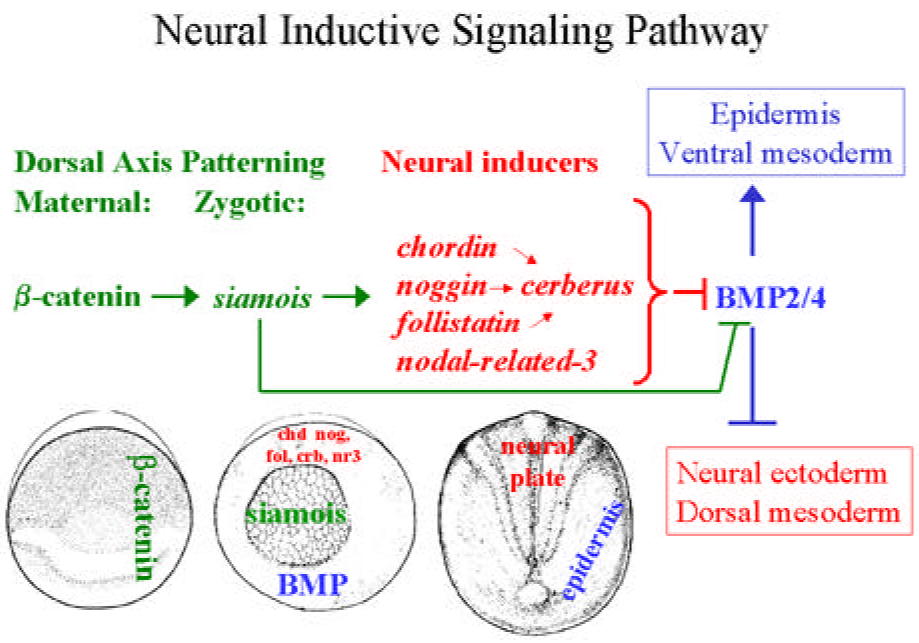

**FIGURE 2.** The neural inductive signaling pathway is diagrammed. Just after fertilization, maternal β-catenin protein is concentrated on the dorsal side of the egg. One of its downstream targets is Siamois, a zygotically transcribed transcription factor expressed in the dorsal endoderm. Siamois activates the transcription of organizer-specific neural-inducer genes (in red) and represses the transcription of BMP2/4 (blue). The neural inducers are secreted proteins that inhibit BMP signaling. BMP signaling promotes epidermal and ventral mesodermal fates, and represses neural ectodermal and dorsal mesodermal fates. Neural ectoderm is formed when BMP signaling is prevented. Drawings of *Xenopus* embryos from [139].

**TABLE 1**
**Names of Neural-Inducing, Anti-BMP Molecules Across Animals**

| | Fly | Frog | Fish | Chick | Mouse | Human |
|---|---|---|---|---|---|---|
| **Chordin** | Short-gastrulation | Chordin | Chordin | Chordin | Chordin<br>Chordin-like<br>Neuralin-1<br>Amnion-less | CHRD<br>LOC57803 |
| **Noggin** | — | Noggin | Noggin 1, 2, 3 | Noggin | Noggin | NOG |
| **Follistatin** | — | Follistatin | Follistatin | Follistatin<br>Flik | Follistatin<br>Follistatin-like<br>Follistatin-like 3<br>Follistatin-related protein<br>Insulin-like growth factor binding protein 7 | FST<br>FSTL1<br>FSTL3<br>SPARC<br>SPARCL1 |
| **Cerberus** | — | Cerberus | — | CCer | Cerberus-related (Cerr1) | CER1 |
| | | Dan | | Gremlin | Cerberus-like (cer-l)<br>Dan<br>Drm (Cktsf1b1)<br>Prdc<br>Dte (dante) | FLJ21195<br>DRM<br>CKTSF1B1 |
| **Nodal-related-3** | — | Xnr-3 | — | — | — | — |

*Note:* Names were retrieved from animal databases where possible (Flybase, Zfin, Mouse Genome Database, LocusLink). The URLs for these sites can be found at http://www.nih.gov/science/models/. Names appearing in parentheses are alternate names. Dashed lines indicate that no gene was found for that organism by searching the literature or the available databases.

Proteins with similar activities have been found in all vertebrate models and *Drosophila* (Table 1). Thus, this mechanism of establishing dorsal-ventral ectodermal fates is evolutionarily conserved[28]. Biochemical analyses demonstrate that Chordin directly binds to BMP2 and BMP4 with high affinity via its cysteine-rich domains, thereby inhibiting signal transduction by competing with BMP receptors[64,100].

Although Chordin is found in most animals, it does not have identical developmental consequences. In amniotes (avians and mammals), for example, Chordin is insufficient to induce neural ectoderm under every condition. In chick, a BMP4/Chordin antagonism appears to exist in the primitive streak region, which is analogous to Spemann's organizer. Ectopic expression of Chordin in the area pellucida generates an ectopic primitive streak and organizer marker genes consistent with the amphibian experiments. But, when Chordin is misexpressed in extraembryonic epiblast, it does not induce neural tissues[118]. Similarly in mouse, Chordin is expressed in the organizer region called the node, but neural induction still occurs in mutant animals that are missing either the node or *chordin*[9,56]. These studies suggest that additional molecules are required to induce neural fate either in some environments, under some experimental conditions, and/or in some organisms. Indeed, there are several other secreted molecules available in the embryo that appear to work, in addition to Chordin, to inhibit BMP signaling and thus promote a neural fate.

In addition to other neural-inducing molecules, recent evidence demonstrates the existence of several molecules that contain similar functional domains to Chordin, and, which therefore may compensate for the loss of *chordin* expression in mutant animals. Mouse Neuralin-1, which can induce a secondary axis in *Xenopus*, is expressed in the neural plate during gastrulation[25]. *Xenopus* Kielin also is expressed in the neural plate. It only weakly induces secondary axes and neural tissues, but strongly activates paraxial mesodermal fates[75]. The mouse *amnionless* gene is required for primitive streak formation and may specify mesodermal fates[52]. Mouse Chordin-like protein induces secondary axes in *Xenopus*, binds BMP4, and is expressed in later developing mesenchymal cell lineages[87]. Each of these is distinct from Chordin in structure, expression pattern, and functional effects, but might substitute for Chordin in experimental embryos or supplement Chordin activity in important ways during normal neural induction.

Noggin, which shares no structural homology with Chordin, also fits all criteria to be a neural-inducing (organizer) molecule. It is expressed in the dorsal lip during gastrulation and later is expressed in the head mesoderm and notochord. In addition to causing the formation of secondary axes, it both dorsalizes mesoderm and induces neural markers in ectodermal explants. Like Chordin, Noggin directly binds to BMP2/4 with high affinity and competes with their receptors. Although the mode of its binding to BMP2/4 is still unknown, certain cysteine residues are required for its biological activity[71]. Noggin does not appear to be expressed in *Drosophila*, but it has been cloned from a large variety of vertebrates. Thus it may be an evolutionary add-on to ensure that neural induction occurs.

Like Chordin, Noggin is expressed in the amniote organizer regions, but alone it is insufficient to cause neural induction. In chick, Noggin is much less efficient than Chordin in inducing an ectopic primitive streak to form neural tissue[116]. Noggin also is insufficient to induce neural ectoderm in the extraembryonic epiblast. In mouse, Noggin is unnecessary for neural induction in node-deleted embryos[56] or *noggin*-null mutants[79]. However, mice in which both *chordin* and *noggin* were deleted showed severe defects in forebrain development[9]. Thus, it is likely that Chordin and Noggin work synergistically to cause formation of the neural ectoderm.

Follistatin is a secreted activin-binding protein that also binds several members of the BMP family. Unlike Noggin and Chordin, Follistatin binds both the BMP ligand and its receptor, forming an inactive trimeric complex[50]. During *Xenopus* gastrulation, *follistatin* is expressed in the dorsal lip and later in the notochord. Like Chordin and Noggin, Follistatin induces secondary axes in embryos and anterior neural markers in ectodermal explants. Chick Follistatin also is expressed in the organizer, and its deletion results in severe forebrain deficits[124]. Incubation of early chick blastoderm in the presence of Follistatin prolongs the tissue's competence for neural induction[124].

Mouse Follistatin is expressed in the organizer region[2,127], but *follistatin*-null mice have no gross neural defects[76].

Cerberus, a member of the DAN family of secreted proteins, also blocks BMP signaling[49]. It is expressed downstream of Noggin, Chordin, and Follistatin in both the head mesoderm part of the dorsal lip and adjacent presumptive head endoderm[15]. Misexpression of *cerberus* on the ventral side of *Xenopus* embryos leads to ectopic head formation, in particular the most anterior structures such as cement gland, olfactory epithelium, and eyes. Unlike Chordin and Noggin, Cerberus also induces cardiac mesoderm and liver. Cerberus specifically binds to BMP2/4, and Noggin can compete with this binding[49]. However, Cerberus also blocks Wnt and Nodal activities by directly binding to them[49,99]. The combined antagonism of both Wnt and BMP family signaling[99] appears to facilitate the strong ability of Cerberus to induce head, rather than trunk, structures.

Cerberus-related molecules play multiple roles in vertebrate development. There are at least five family members in the mouse, only some of which can induce neural ectoderm[12,94]. Single gene knockouts of two family members most related to Cerberus do not cause the anterior neural defects[12,113], suggesting that either Cerberus-related molecules are unnecessary or that the different family members compensate for each other. Although all tested mouse Cerberus-related proteins inhibit BMP signaling, not all suppress Wnt signaling[12,94]. The only reported chick Cerberus-related protein has been implicated in left-right asymmetry of head structures rather than neural induction[138]. However, it is likely that there are other Cerberus-related molecules in chick that have yet to be identified. The functional variation within the Cerberus-related family of BMP antagonists is not fully understood, and needs to be further elucidated in order to understand their roles in different developmental processes, including neural induction.

Nodal is a TGF-β family member expressed in the mouse node and required for axis-formation[128]. In all vertebrates studied there are multiple nodal-related (Nr) proteins that regulate a number of patterning events. In *Xenopus* there are at least six Nr proteins, all but one of which strongly induce mesoderm and endoderm[91]. The exception is Xnr3, which is expressed in the superficial ectodermal cells of the organizer, rather than the involuting mesoderm, in response to maternal β-catenin signaling[46,78]. It does not induce mesoderm, but directly induces neural ectoderm. Xnr3 is different from the above neural-inducing proteins in that although it functions by inhibiting BMP4 signaling, there is no evidence that it directly interacts with the BMP4 ligand[33]. There is no information about this gene in other vertebrates.

The fact that the embryo uses at least five different gene products, all of which act by inhibiting BMP signaling, to form the neural ectoderm indicates the necessity for turning off this pathway. The requirement to inactivate BMP signaling on the dorsal side is further emphasized by the expression of several factors in the organizer that also repress BMP transcription[10,21,38,39,68]. Thus, to achieve neural induction the ligand must be inactivated, and transcription must be down-regulated. In *Xenopus*, inhibition of the ligand appears sufficient to cause embryonic ectoderm to become neural. Ectopic expression of a single BMP antagonist causes secondary axes to form and induces neural tissues in ectodermal explants. But studies in chick and mouse indicate that other signaling pathways are involved as well. As mentioned above, knockout of some of the BMP-binding genes or deleting the organizer does not completely delete neural structures, although some defects are apparent in some mutants. These results can be explained by either a preponderance of compensatory molecules or the necessity for additional signals. We know the former to be the case, as described above, and there also is compelling evidence for the latter.

It is well documented that FGF family members play an important role in establishing the posterior axis of the neural plate (Fig. 1[36,42]). In addition, both frog and chick experiments indicate that FGFs have neural inducing activity[51, reviewed in 117,134]. In explants, Chordin and Noggin require intact FGF signaling to induce neural tissue, although this is not so in whole embryos. One explanation for the latter result is that there may be a receptor-specificity requirement[48]. In chick, FGF3 is expressed in the medial epiblast that will become the neural plate. High levels of FGF3 signaling appear to repress BMP transcription and low levels appear to promote a neural fate.

However, FGF3 also is expressed in lateral regions of the chick epiblast that also express BMP and an epidermal fate. Because lateral epiblast cells resist neural induction by FGF3 or BMP antagonists, another antineural signal must be present[134]. Several experiments indicate that members of the Wnt family antagonize the neural-promoting effects of FGFs. Thus, a current hypothesis is that neural induction occurs via an early FGF signal, in a Wnt-free environment, that both represses BMP transcription and promotes a neural fate. The former action is strongly reinforced by the expression of BMP antagonists. However, the characteristics of the latter are not yet elucidated. An additional insufficiently explored mystery is what establishes the differential regions of FGF and Wnt signaling. There is evidence in frog, chick, zebrafish, and mouse that an ectodermal prepattern exists prior to the overt formation of the respective organizer structures[117,120,134,133]. As described in the following section, many neural–fate-stabilizing genes are expressed maternally in precursors of the dorsal ectoderm. Understanding the establishment of this prepattern is an important key to understanding the molecular mechanisms that will transform ES cells into neural progenitors.

Directing naïve stem cells to a neural fate is a stem cell biology challenge. Scientists know that both human and mouse embryonic stem cell lines can make limited numbers of neurons, but these cultures are predominantly a mixture of many other cell types[44,89,112]. Two treatments have had moderate success in enriching mouse ES cultures in differentiated neurons: cultivation of ES cells as aggregates (embryoid bodies) and treatment of cultures with RA[44,119]. More recently, two feeder cell substrates secreting yet-to-be defined factors have been reported to increase the yield of neuronal derivatives of ES cultures [53,102]. The molecular mechanisms by which these results are achieved are unknown, so the question of how one can direct ES cells to a predominantly or purely neuronal stem cell fate remains. Based on the developmental work presented above, it would seem that this could be accomplished simply by inhibiting BMP signaling at the early stages of ES cell cultures. A few studies have begun to investigate this possibility. Treatment of embryoid bodies with BMP4 significantly reduced the number of neural cells in a dose-dependent manner[35]. More recently, Tropepe et al.[125] demonstrated that growing ES cells at low density without a feeder cell layer caused them to readily acquire a neural stem cell identity. Previously, the same effect had been shown in cultured amphibian embryonic ectoderm[43,109]. The accepted explanation is that this prevents a pericellular accumulation of BMP, thus activating the neural "default" state in these cells. Although application of Noggin to conventionally acquired embryoid bodies did not induce neural fates[35], both Noggin and Cerberus treatment of low-density ES cells significantly increased the formation of neural stem cell colonies[125]. Interestingly, these effects require the continuous presence of FGFs, perhaps to reduce endogenous BMP transcription. BMP antagonism appears to be important for the production of neural stem cells within the adult brain. Noggin is secreted by the ependymal cells, which are adjacent to the cortical stem cells in the subventricular layer[70]. Exogenous Noggin applied to this region promotes neuronal differentiation whereas exogenous BMP promotes glial differentiation. This study suggests that *in vivo* replacement of damaged tissue by endogenous neural stem cells might be accomplished by manipulating the levels of BMP antagonists.

These studies have helped scientists understand how to use the information gleaned from the embryo to manipulate ES cells to a neural fate, but much more work needs to be done. In particular we need to know which combinations of neural-inducing molecules will have the desired effect and whether these molecules are interchangeable and functionally redundant. Or, do they have discrete effects that might bias primitive neural stem cells in subtle but significant ways? It will be very important to elucidate the relative roles of all of those named above, and possibly new, players in embryonic neural induction so we can better understand how to convert ES cells, embryonic ectoderm, and even adult neural tissues to neurally committed stem cells in reliable and efficient ways.

## Neural Fate Stabilization

An important and understudied step of the neural specification program outlined in Fig. 1 is the stabilization of neural fate that occurs as the presumptive neural ectoderm is consolidated into a *bona fide* neural plate. Vertebrates possess several genes that are transcribed between neural induction and the onset of differentiation (proneural) gene expression[108]. Some of these intermediate genes appear to modulate competence of neural ectoderm to respond to signaling molecules (*sox*, *zic*); some appear to hold neuroectodermal cells in a state of commitment to a neural fate while maintaining a proliferative capacity (*geminin*) or inhibiting differentiation (*foxD5*, *zic2*); others promote differentiation pathways upstream of proneural genes (*soxD*, *Xiro*). These "neural fate-stabilizing" genes are important for the process of expanding neurally committed, undifferentiated embryonic stem cells. As such, they are important candidates for potential manipulation to expand both ES and adult stem cells along a neurally committed pathway (Table 2).

### *Neural Fate-Stabilizing Genes That Promote Neural Ectodermal Competence*

Competence is the ability of an embryonic tissue to respond to signaling factors. Members of both the *sox* (Sry-related transcription factors that contain an HMG DNA-binding domain) and *zic* (zinc finger containing factors with significant homology to *Drosophila odd-paired*) transcription factor families appear to promote a neural fate by enhancing the embryonic ectodermal response to neural inducers and/or stabilizing that response.

Three *sox* genes (*sox1*, *2*, *3*) are expressed in the early neural ectoderm of many vertebrates[131]. All three are expressed throughout the entire presumptive neural ectoderm prior to the onset of gastrulation, and soon after they become highly expressed in the neural plate, cranial placodes, and neural crest[27,96,103,126,136]. These genes also are expressed in some non-neural tissues, the best studied of which is the lens. There is a paucity of functional studies of *sox1* in the nervous system, although one study suggests an early role in neural specification because Sox1 can substitute for RA in inducing P19 embryonal carcinoma cells to express a neural fate[98]. In *Xenopus*,

**TABLE 2**
**Names of Neural Fate-Stabilizing Genes Across Animals**

| | Fly | Frog | Fish | Chick | Mouse | Human |
|---|---|---|---|---|---|---|
| **SOX 1-3** | *soxN* | *sox1*<br>*sox2*<br>*sox3* | *sox3* | *Sox1*<br>*Sox2*<br>*Sox3* | *Sox1*<br>*Sox2*<br>*Sox3* | *LOC137208*<br>*(sox2)* |
| **Zic 1-3** | — | *zic1 (opl)*<br>*zic2*<br>*zic3* | *zic1 (opl)*<br>*zic2* | — | *Zic1*<br>*Zic2*<br>*Zic3* | *ZIC1*<br>*ZIC2*<br>*ZIC3* |
| **Geminin** | *geminin* | *geminin* | — | — | *Geminin* | *LOC51053* |
| **FoxD5** | — | *foxD5* | *foxD5*<br>*(fkd8)*<br>*(forkhead-8)* | — | — | — |
| **SOXD** | — | *soxD* | — | | — | — |
| **Iroquois** | *mirror*<br>*caupolican*<br>*araucan* | *Xiro1*<br>*Xiro2*<br>*Xiro3* | *iro1*<br>*iro3* | *Irx2*<br>*Irx3* | *Irx1*<br>*Irx2*<br>*Irx3* | *IRX1*<br>*IRX2*<br>*IRX3* |

*Note*: Names were retrieved from animal databases where possible (Flybase, Zfin, Mouse Genome Database, LocusLink). The URLs for these sites can be found at http://www.nih.gov/science/models/. Genes appearing in parentheses are former names. Dashed lines indicate that no gene was found for that organism by searching the literature or the available gene databases.

*sox2* and *sox3* are activated by the inhibition of BMP, either by expression of BMP antagonists, or by dissociation of embryonic ectoderm[82,96]. Ectopic expression of *sox2* cannot induce neural markers on its own, but can do so when expressed in combination with bFGF[96]. These results suggest that *sox2* is involved in changing the responsiveness of the neural ectodermal cells to FGF signaling. It is not known which aspect of FGF signaling, neural induction, or anterior-posterior axis formation, is affected by *sox2*. Interestingly, Sox2 has been shown to activate the *fgf4* gene by its DNA bending activity[110]. *sox2* is apparently essential for the further differentiation of the neural ectoderm; when it is inhibited by the expression of a dominant-negative construct, the neural ectoderm does not express later markers such as *N-CAM*, *Xngnr1,* and *n-tubulin*[55]. Earlier-expressed neural plate markers are not inhibited by this construct, nor are epidermal markers induced, indicating that *sox2* is not involved in the fate choice between neural vs. epidermis, but is necessary for the induced neural ectoderm to begin to differentiate.

*sox1* and *sox2* are expressed by undifferentiated neural precursor cells in RA-induced ES cell cultures[69]. Li et al.[69] also showed that the *sox2* promoter driving a neomycin resistance gene could be used to select neural progenitors from these cultures. Zappone et al.[137] identified an upstream region of the *sox2* promoter that drives expression in the ventricular zone of the telencephalon where neural stem cells reside. They showed that telencephalic cells expressing a *sox2*-promoter transgene divided in culture for several months longer than nontransfected cells. This result suggests that those factors controlling the telencephalic expression of *sox2* play important roles in maintaining the neural-specified stem cell state.

*sox3* is highly related to *sox1* and *sox2*. It shares very similar expression patterns with *sox2* in most vertebrates. However in *Xenopus* it also is expressed during oogenesis[60], and after fertilization the maternal transcripts are localized to the animal hemisphere that gives rise to the embryonic ectoderm[96]. The maternal function of *sox*3 is unknown, and there are very few studies of *sox3* zygotic function, but it has been proposed that Sox1, 2, and 3 can substitute functionally for one another in the neural plate[82]. A few studies have investigated the role of *sox3* in cranial placodes. In the chick, *sox3* is expressed upstream of proneural differentiation genes, and its overexpression interferes with the migration of differentiating neurons from the placodes[1]. In medaka fish, overexpression of *sox 3* causes ectopic placode structures to form[59]. Although these studies suggest that *sox3* plays an intermediate role in peripheral neuronal specification, more experiments need to be done to elucidate the embryonic function of *sox3* in neural stem cell development.

The *zic* genes were first isolated in the mouse because of their high levels of expression during cerebellar development [6,7,8]. *zic1, 2,* and *3* have very similar expression patterns in mouse, fish, and frog[62,82,84,85,86,104]. They are broadly expressed in the presumptive neural ectoderm during early gastrulation and at late gastrula stages they become restricted to the lateral margins of the anterior neural plate. At later stages they are expressed in the dorsal neural folds/tube, predominantly in the head rather than trunk regions, and in some non-neural tissues. The extinction of *zic* expression along the neural plate midline and ventral neural tube appears to be under the control of *sonic hedgehog*[84,104].

In *Xenopus*, *zic1* and *zic3* are transcribed 30–60 min after the *chordin* gene and can be induced in animal caps by BMP antagonism[62,82,85,86]. An elegant series of animal cap explants indicate that *zic1* expression (also called *opl*) allows ectoderm to be more sensitive to neural induction by Noggin[62]. Overexpression of *zic1* and *zic3* expands the neural plate and crest, concomitantly represses epidermal fate, and induces the expression of a number of proneural genes. Together, these studies indicate that *zic1* and *zic3* first mediate neural competence, promoting the progression to differentiation, and later promote neural crest fate. The function of *zic2* appears to be somewhat different from *zic1* and *zic3* and is discussed in the next section. However, all three genes have some level of functional redundancy because of the very similar phenotypes after gene overexpression and because *zic1* and *zic2* knockout mice show normal early neural development. However, they do not have identical functions, as indicated by the differing cerebellar defects in the knockout mice[5] and the different defects caused by mutations in the human homologues[17,37].

## *Neural Fate-Stabilizing Genes That Antagonize Differentiation*

Other neural fate-stabilizing genes function between neural induction and neuronal differentiation to either influence the proliferative activity of neural plate stem cells (*geminin*) and/or inhibit their differentiation (*foxD5, zic2*).

Geminin is a 25-kDa cytoplasmic protein that inhibits DNA replication by interacting with Cdt1, a replication initiation factor needed to load the mini-chromosome maintenance complex to replication origins[77,123,135]. *geminin* was first cloned in *Xenopus* as a maternally expressed transcript localized to the animal hemisphere of cleavage embryos, which is the region that gives rise to the future neural ectoderm[61]. During gastrulation, *geminin* is re-expressed as a zygotic transcript in the dorsal ectoderm. Later it is expressed in the anterior domain of the neural plate and then restricted to the floor and roof plates of the neural tube. The onset of *geminin* zygotic transcription occurs after the organizer genes start to be expressed, and Noggin and Chordin induce *geminin* expression in animal cap explants. Thus, Geminin acts downstream of neural induction. Ectopic expression of *geminin* mRNA expands the neural plate and induces early neural differentiation genes. Likewise, overexpression of *Drosophila* Geminin induces ectopic neural differentiation[101]. Although overexpression of Geminin inhibits DNA replication, during normal development the protein is present in cycling cells, accumulating during S phase and being degraded at the metaphase-anaphase transition[101]. *Drosophila* embryos carrying a null mutation in *geminin* show increased numbers of S-phase cells. These data suggest that geminin keeps cells cycling during neural plate stages, which allows for the expansion of these early neural stem cells.

*foxD5* is a member of the *forkhead/winged helix* family of transcription factors cloned from *Xenopus*[34,115,121]. *foxD5* transcripts are first detected during oogenesis and become localized to the animal hemisphere that gives rise to the embryonic ectoderm. Zygotic expression begins just before gastrulation throughout the presumptive neural ectoderm. It is maintained in the neural plate and becomes extinguished as the neural folds elevate and fuse. Expression of *foxD5* in animal cap explants induces elongation and expression of some mesodermal, neural-inducing, and early neural-specifying genes, indicating a role in dorsal axis formation. Zygotic *foxD5* expression is induced strongly by Siamois, moderately by Cerberus, weakly by Wnt8 and Noggin, and not by Chordin in animal cap explants. Together these experiments suggest that FoxD5 affects neural fate in a pathway parallel to Noggin-Chordin mediated neural induction (Fig. 3). However, it cannot independently induce neural tissues from epidermis in whole embryos, suggesting that it regulates genes just downstream of BMP antagonism. In contrast, the overexpression of *foxD5* in the neural ectoderm causes hypertrophy of the neural plate and expansion of early neural genes (*sox3* and *otx2).* This does not result from increased proliferation or expanded neural-inducing mesoderm. Instead, the neural plate is maintained in an immature state because *otx2* expression is expanded, and *en2, Krox20,* proneural, and a neural differentiation gene (*n-tubulin*) expression are repressed. Expression of *foxD5* constructs fused with the *engrailed* repressor domain or with the VP16 activation domain demonstrates that FoxD5 acts as a transcriptional repressor in axis formation and neural plate expansion. Deletion constructs indicate that this activity requires the C-terminal domain of the protein, which contains a charged amino acid-rich region. Together these data indicate that *foxD5* functions to expand the already induced neural ectoderm, much like the *sox* genes, and to maintain this ectoderm in an undifferentiated state via the repression of axial patterning and differentiation genes[121]. Retained in an immature state, the neural ectoderm can expand and be refractory to signaling that might push the cells towards differentiation.

Several other *forkhead* genes also are expressed in the *Xenopus* neural ectoderm and plate[58]. However, they likely function differently from *foxD5*. First, unlike *foxD5,* most are induced strongly by BMP4 antagonists. Second, they are expressed in smaller domains of the neural plate. *XFD1/XFKH1/pintallavis* (*foxA4*) and *XFKH5* (*fox* B class) are expressed only in the floor plate[30,31,57,105], *XFKH4/XBF1* (*fox* G class) and *XBF2* (*fox* D class) are expressed predominantly in the anterior neural plate[14,31,74], and *XFKH6* (*foxD3*) is expressed along the lateral border of

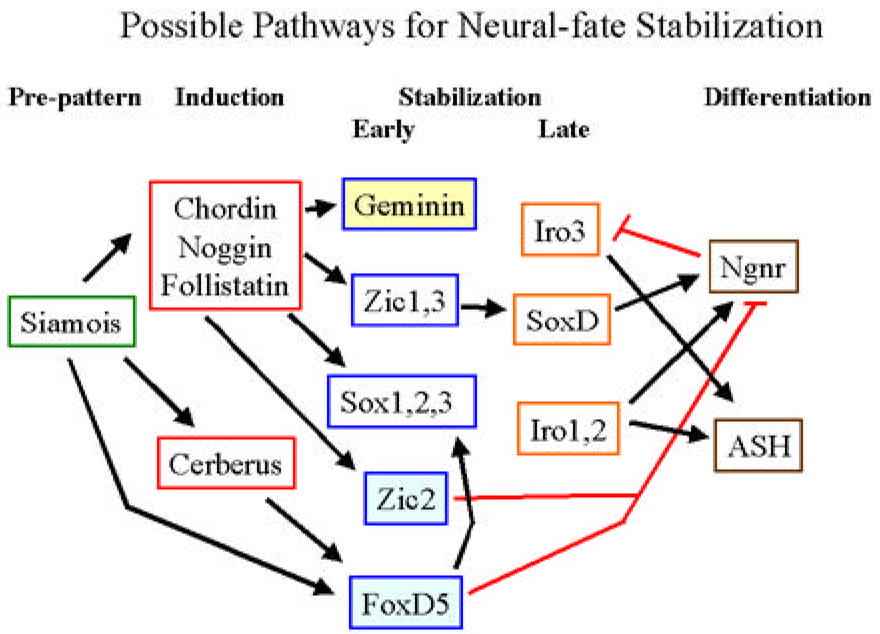

**FIGURE 3.** Many genes are involved in the neural fate stabilization pathway. Arrows indicate experimental evidence (reviewed in text) that the upstream gene activates the expression of the downstream gene. Red bars indicate experimental evidence that one gene inhibits the expression of another. Arrows and bars are missing at many levels because the data are yet to be reported. All neural fate stabilization genes (blue and orange boxes) are downstream of neural inducers (boxed in red). However, FoxD5 is not downstream of Chordin or Noggin, but is induced by the Siamois/Cerberus pathway. All neural fate stabilization genes are upstream of early expressed proneural genes (boxed in brown) and the later differentiation pathway. Those neural fate stabilization genes that are expressed earliest in the neural plate are boxed in blue, and those that are expressed later are boxed in orange. The early neural-fate stabilizing genes either enhance differentiation (white shading), inhibit differentiation (blue shading), or promote proliferation (yellow shading). The late neural-fate stabilizing genes are thought to be just upstream of early proneural genes. There is a paucity of knowledge of how the many different neural fate-stabilizing genes regulate each other and their downstream targets.

the midbrain/hindbrain region[31,67]. In contrast, *foxD5* is expressed broadly throughout most of the neural plate but only for the limited time during which it is expanding. Analyses of function have been reported for only a few of these genes. *XFD1* represses anterior neural structures and expands posterior neural tissue[105]. *foxD3* regulates neural crest formation downstream of *sox2*, and perhaps in cooperation with Zic factors[107]. *XBF1*, *XBF2*, and *foxD5* all expand the neural plate and repress differentiation. But, whereas *foxD5* expands an anterior character (*otx2*) in the neural plate and represses more posterior markers (*en2*, *Krox20*), *XBF2* induces both anterior and posterior neural markers. *foxD5* does not directly convert ectoderm to a neural fate, whereas *XBF2* does. *foxD5* represses differentiation, whereas *XBF1* is posited to act in the early steps of neuronal differentiation. Subtle variations in function of these several *fox* (*fork head*) family members in the neural ectoderm may regulate discrete processes leading from the acquisition of neural competence to full neural differentiation. The elucidation of these processes and the role of Fox proteins in them will be key to understanding the translation of neural inductive signaling into the mature patterning and differentiation of the nervous system.

In general, *zic2* is expressed in a similar pattern to *zic1* and *zic3*[16,86]. However, when *zic1* and *zic3* become restricted to the neural plate during gastrulation, *zic2* remains expressed throughout the dorsal ectoderm. Also, *zic2* expression remains in the posterior part of the neural plate as the other two genes become anteriorly restricted. Interestingly, this posterior expression is in longitudinal stripes that alternate with the stripes of proneural-expressing, i.e., differentiating, cells. Finally, *zic2* is the only one of the three that is expressed maternally[86]. As reported above for *zic1* and *zic3*, overexpression of *zic2* results in the expansion of the neural plate and crest, and the reduction of epidermal markers. However, closer inspection demonstrated that, like *foxD5*, this expansion is accompanied by repression of neural differentiation markers[16]. In fact, *zic2* can counteract the formation of ectopic neurons produced by either *neurogenin* or *Gli1/2* mRNA injections. Like FoxD5, Zic2 contains mono-amino acid stretches that are suggestive of a repressor function. The fact that a VP16-Zic2 transcriptional activator construct produced phenotypes opposite from the wild type protein agrees with this assessment. Brewster and Ruiz i Altaba[16] propose that the early function of *zic2* is to negatively regulate the domains of neural stem cells that can initiate differentiation. They speculate that its later function to promote neural crest fate may also be mediated by repression of bHLH and Gli factors.

## Neural Fate-Stabilizing Genes That Promote Differentiation

A few neural fate-stabilizing genes (*soxD*, *Xiro*) also expand the neural ectoderm, but in addition they appear to promote the initiation of differentiation by acting just upstream of the proneural genes. As such, they may act in opposition to *foxD5* and *zic2*.

*soxD* is distantly related to *sox2* and *sox3*[81]. It is expressed widely in the presumptive neural ectoderm, but by late gastrulation its midline expression is extinguished, much like the *zic* genes. In animal cap explants it is induced by BMP antagonism and by Zic1. Ectopic expression of *soxD* resulted in ectopic neural masses in embryos and induced both anterior neural markers and early proneural genes. Inhibition of *soxD* by a dominant-negative construct suppressed anterior neural tissues, which could not be rescued by the expression of *sox2*. These results indicate that the early expression of *soxD* may be involved in neural competence, like the other *sox* genes and *zic* genes, but probably acts downstream of them. Later, *soxD* appears to specify the forebrain region by positively regulating proneural genes.

Homologues of genes within the *Drosophila Iroquois* complex (*ara* and *caup*) have been cloned in mouse[13,26,83,97], chick[41,90], *Xenopus*[11,40], zebrafish[23,130], and human[90]. The *Drosophila Iroquois* genes encode homeodomain proteins that are required for the activation of *achaete* and *scute*[22]. *Xenopus iro1* and *2 (Xiro1,2)* are expressed in the entire presumptive neural plate at the beginning of gastrulation, earlier than the three earliest-expressed proneural genes (*XASH3*, *XATH3,* and *Xngnr1*), in regions that overlap with, but are broader than, the proneural gene expression domains. Later, *Xiro1,2* are expressed only in dorsolateral neural plate posterior to *otx2* expression and in the neural crest ectoderm. *Xiro3* is expressed a little later, at mid-gastrulation, in the same later pattern as *Xiro1,2*. *Xiro3* expression precedes those of *XASH3* and *XATH3,* but not *Xngnr1*. All three *Xiro* genes are induced only weakly, if at all, by Noggin. However, treatment of Noggin-induced caps with RA or Gli1 greatly enhances *Xiro1,2* expression, but not that of *Xiro3*. Treatment of Noggin-induced caps with FGF greatly enhances *Xiro3* expression. These results are consistent with the later posterior restricted expression domains of these genes. It has been additionally shown that *Xiro1* expression requires Wnt signaling and that Xiro1 and BMP4 are mutually repressive[38,39]. The overexpression of all three genes causes the neural plate to be expanded, with a decrease in neural crest markers, which as mentioned earlier are negatively regulated by proneural genes. *Xiro1,2* greatly expand *XASH3* and *Xngnr1* expression. *Xiro3* also expands *XASH3* expression in the neural plate, but not outside the nervous system. *Xiro3* does not induce *Xngnr1*, but *Xngnr1* represses *Xiro3* expression. Further, the later neuronal differentiation marker, *n-tubulin*, is repressed by *Xiro3*. Although many more experiments are needed to fill out this story, it appears that *Xiro1,2* function to upregulate the earliest proneural genes (*Xngnr1*, *XASH3*) to promote the onset of neural differentiation. *Xiro3* is expressed slightly later, downstream of *Xngnr1*, to promote *XASH3* expression but prevent overt differentiation. By working in concert, these genes may precisely regulate the temporal and spatial transition between neural stem cell expansion and the earliest steps in differentiation. Experimental data on the function of other vertebrate *Iroquois* genes are lacking at this time.

## How Might These Genes Regulate Neural Stem Cells?

The studies reviewed above demonstrate that during normal embryonic development there are several major steps in the specification of naïve embryonic cells to differentiated cell types (Fig. 1). It is clear that each of these steps is a multifaceted process involving cell-cell signaling and transcriptional regulation. In the past decade, enormous progress has been made in elucidating these steps in vertebrate neurogenesis, but there are still many gaps in our knowledge that could clarify how to manipulate ES cells to neural stem cells. We do not know the role of maternal factors in biasing the dorsal ectoderm to respond to neural inductive signals[120]. Many of the factors involved in neural fate stabilization, for example, are maternally expressed in the cleavage stage precursors of the neural ectoderm in amphibians. It will be important to know if they influence the responsiveness of embryonic ectoderm or ES cells to neural-inducing factors. We also do not know all of the factors involved in each of the steps; there are likely to be many more players involved in neural induction and neural fate stabilization whose roles in these processes need to be understood. Finally, we need to know how the various known transcription factors relate to one another (Fig. 3). The functions of the genes described remain undistinguished. They all expand the neural plate and several of the same neural markers, but we do not know how they are related to one another or whether they are arranged in a gene pathway or multipath hierarchy. What are their transcriptional targets and signaling molecules? In the next decade, the combined approach of genomics, genetics, and developmental biological manipulations[111] should reveal many of these answers.

There is great excitement in the field of stem cell biology because it may enable at-will production of specific kinds of neural cells from ES cells, adult brain, and even bone marrow. But so far this blossoming field has mostly utilized a trial-and-error approach to coaxing cell sources to express neuronal phenotypes. Although this has been a fruitful approach, it is exciting that researchers are now beginning to apply the information obtained from studies of normal neurogenesis to the production and differentiation of neural stem cells[102]. How will the considerable knowledge base

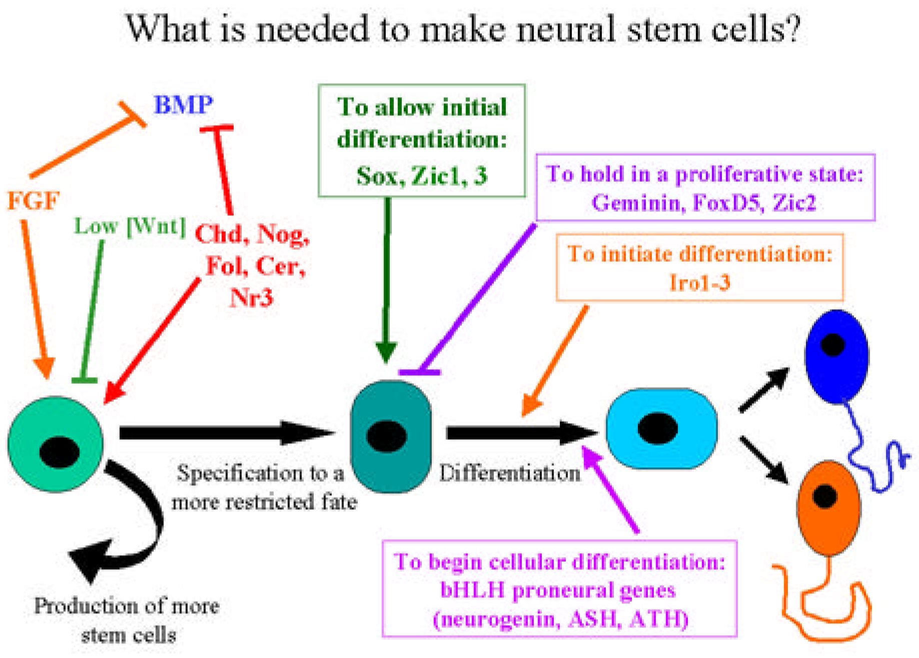

**FIGURE 4.** A proposed scheme for what genes might need to be activated, based on the study of normal neurogenesis reviewed in this article, to produce neural-fated stem cells and to expand those cells prior to differentiation.

about the normal processes of neural specification derived from the several animal models correlate to the goals of obtaining large numbers of cells that will express only a neural fate when transplanted under clinical conditions? First, the process of neural induction needs to be fully elucidated. Experiments so far indicate that simply exposing ES cells to anti-BMP secreted proteins will not be sufficient, although regulation of the downstream events of BMP transcription and signaling are critical. However, we also need to know how these events interact with other signaling systems, in particular FGFs and Wnts (Fig. 4). This information could lead to culturing protocols that greatly enhance the proportion of neurally fated stem cells in ES cell cultures. Second, the regulation of the process by which the neurally induced ectoderm is converted to a neural fate stabilized, proliferative population needs to be understood in detail. A large number of factors have been identified (Fig. 3) that all can expand the neural ectoderm, yet they appear to have subtly different roles in controlling whether the stem cell population remains proliferative or begins to differentiate. We need to understand how these factors interact with each other and with environmental signals to define better the process of neural fate stabilization and be able to drive neural stem cells down the desired developmental pathway (Fig. 4). Once the precise function of these genes is elucidated in animal models, we may be able to regulate their expression, e.g., via transgenic approaches, to expand neural stem cells, define various stages of neural stem cell commitment, and put this information to clinical use.

## ACKNOWLEDGEMENTS

We wish to thank the members of the Moody lab for their helpful comments. Some of the research presented in this article was supported by NIH grant NS23158.
